# Changes in trends and patterns of glycaemic control at Ghana’s National Diabetes Management and Research Centre during the era of the COVID-19 pandemic

**DOI:** 10.1371/journal.pgph.0002024

**Published:** 2023-06-14

**Authors:** Swithin Mustapha Swaray, John Tetteh, Sampson Kafui Djonor, George Ekem-Ferguson, Ruth Yawa Clottey, Atiase Yacoba, Alfred Edwin Yawson

**Affiliations:** 1 National Cardiothoracic Centre, Korle Bu Teaching Hospital, Accra, Ghana; 2 Department of Community Health, University of Ghana Medical School, College of Health Sciences, University of Ghana, Accra, Ghana; 3 Central Laboratory Services, Korle Bu Teaching Hospital, Accra, Ghana; 4 Department of Psychiatry, Korle Bu Teaching Hospital, Accra, Ghana; 5 National Diabetes Management and Research Centre, Korle-Bu Teaching Hospital, Accra, Ghana; 6 Department of Medicine & Therapeutics, University of Ghana Medical School, Accra, Ghana; University of Embu, KENYA

## Abstract

**Background:**

Maintaining optimal glycaemic control (GC) delays the onset and progression of diabetes-related complications, especially microvascular complications. We aimed to establish the trend and pattern of GC, and its associated factors in persons living with diabetes (PLWD), and to examine the influence of COVID-19 on GC.

**Methods:**

A retrospective study involving secondary data from 2,593 patients’ physical records from the National Diabetes Management and Research Centre (NDMRC) in Accra, extracted from 2015–2021. Growth rate of GC was assessed, and ordinal logistic and Poisson models weighted with Mahalanobis distance matching within propensity caliper were adopted to assess the impact of COVID-19 pandemic on GC. Stata 16.1 was utilized and the significant value set as p≤0.05.

**Results:**

GC pattern indicated a steady deterioration ranging from 38.6% (95%CI = 34.5–42.9) in 2015 to 69.2% (95%CI = 63.5–74.4) in 2021. The overall growth from 2015–2021 was 8.7%. Being a woman and increasing diastolic pressure significantly increase the likelihood of poor glycaemic control (PGC) by 22% and 25%, respectively compared with their respective counterparts [aOR(95%CI = 1.01–1.46 and 1.25(1.10–1.41), respectively]; whilst lower age increased the risk of PGC throughout the years. We found that risk of PGC during the era of COVID-19 was approximately 1.57(95%CI = 1.08–2.30) times significant, whilst the adjusted prevalence ratio (aPR) of PGC during the era of COVID-19 was approximately 64% significantly higher than the era without COVID-19 (aPR = 1.64, 95%CI = 1.10–2.43).

**Conclusion:**

GC worsened from 2015–2021, especially during the COVID era. Younger age, uncontrolled blood pressure and/or being a woman were associated with PGC. The NDMRC and other centres that provide specialist healthcare in resource-limited settings, must determine the factors that militate against optimal service delivery in the era of the COVID-19 pandemic, and implement measures that would improve resilience in provision of essential care in the face of shocks.

## Introduction

In recent times, diabetes has become unenviably the gravest life-threatening Non-Communicable Disease (NCD) with prevalence reaching pandemic proportions [1]. With 6.7million deaths attributable to diabetes in 2021, the International Diabetes Federation (IDF) estimates 537 million adults aged 20 to 79 are living with the condition; and by 2030, this number is expected to increase to 643 million, and to 783 million by 2045 [1, 2]. Though with the least prevalence (4.5%), Africa currently records the highest proportion of undiagnosed diabetes amongst all IDF regions at 54% [2]. Each year, about three-quarters of all diabetes-related deaths occur in adults under the age of 60, the greatest proportion of any age group in the world [2]. Estimates put Ghanaians living with diagnosed diabetes at 281,000 with an overall adult population prevalence rate of 6.46% [3]. With global trends showing a steady rise in incidence over the years, it is very likely current estimates would be significantly higher, especially with the high rate of undiagnosed diabetes.

Diabetes is a long-term condition that impairs the pancreas’ ability to generate insulin as well as how the body uses it [4]. It is induced by genetic predisposition along with environmental influences [5]. The condition can be difficult to manage and control. However, the salutary rewards are healthy longevity and improved quality of life with adequate glycaemic regulation [6]. In Ghana, many people (59.4%) with diabetes are hardly making treatment progress due to poorly controlled blood glucose even with glycated haemoglobin (HbA1c) cut-off of 8.0% for good control [7]. Based on data collected in Aschner et al’s [8] large international observational study involving over 66,000 persons with type 2 diabetes, glycaemic control in developing nations remained suboptimal over 12 years spanning 2005 to 2017. And despite pharmacological advancements in the field of diabetes care, glycaemic control continues to rapidly worsen [8]. This phenomenon is not peculiar to the African continent. The estimated prevalence of diabetes in American adults grew dramatically between 1999–2000 and 2017–2018, with only about 21% of persons with diagnosed diabetes achieving all three risk factor management objectives (HbA1c < 7.0% or individualized HbA_1c_ targets, BP < 130/80 mm Hg and LDLc < 100 mg/dL) in 2015–2018 [9].

A major concern of diabetes is the accompanying complications, which occur as detrimental consequences of hyperglycaemia [10]. The disease is strongly associated with early onset of micro and macrovascular complications as well as reduced quality of life [11]. Poor glycaemic control (PGC) can lead to an increased risk of blindness, end-stage renal disease, cardiovascular disease, and lower-limb amputations [12]. Preventing the harmful effects of hyperglycaemia is the most important part of the appropriate management for persons living with diabetes (PLWD). As a result, rigorous glycaemic management is regarded as the primary therapeutic target for averting these complications and improving quality of life [10, 13]. In support, the Diabetes Standards of Care 2022 [14] recommends timely treatment decisions that rely on evidence-based guidelines, considering individual preferences as well as social/community support, without neglecting co-morbidities, prognosis, and financial implications for patients.

In the wake of the COVID-19 pandemic, the vulnerability of people with diabetes, especially those with uncontrolled blood glucose and/or having other comorbidities has become evident owing to their increased risk of disease severity and death [15]. In the initial stage, the focus on containing the impact of the COVID-19 pandemic led to disruption in other essential healthcare services in 90% of countries globally. A WHO report cited diabetes as one of the most extensively affected health services interrupted as a result of countries having to make radical decisions in responding to the pandemic [16]. Despite reduction in COVID-19 incidences and restrictions, assessing the impact of the pandemic on diabetes trends may influence strict protocols and public health management interventions in curtailing the disease and subsequent others of similar nature. Additionally, this study was conducted at the National Diabetes Management and Research Centre (NDMRC) of Ghana to assess determinants of glycaemic control in a long-term duration. The study, therefore, examined the trends and patterns of glycaemic control, and the influence of COVID-19 on these trends whilst making some projections into the next few years ahead.

## Methods

### Study Setting

This study was conducted at the National Diabetes Management and Research Centre (NDMRC) in Korle-Bu, Ghana. The facility was founded in 1995 as a diabetes treatment, research, and training centre of excellence. It is part of the Department of Medicine and Therapeutics of the Korle-bu Teaching Hospital. The centre is currently being remodeled to make it more accommodating for its customers and the rising number of persons with diabetes. With the addition of a laboratory, pharmacy, and physical therapy unit, the NDMRC is ready to become a multiservice Centre for its clients. It has more than 5000 registered users and serves as an ideal environment for unrestricted diabetes research. The facility trains both undergraduate and postgraduate medical professionals to manage diabetes as part of its training mandate. An average of 80 clients are seen daily from Monday through Friday. The NDMRC operates on ambulatory/outpatient basis. Services include dietherapy, ophthalmology, and psychological support. Patients’ progress is monitored periodically, usually quarterly or biannual using laboratory tests such as HbA_1C_, renal function test, lipid profile, full blood count and urinalysis. Patients have an individualized management plan.

### The healthcare system of Ghana and COVID-19 pandemic measures

In line with Ghana’s universal health care system, the main healthcare model of the Korle-Bu Teaching Hospital and for that matter, the NDMRC is to a large extent hinged on the national health insurance scheme.

The country had its fair share of the havoc wrecked by the COVID-19 pandemic. The country experienced a three-week partial lockdown in two of its most populous and economically vibrant cities (Greater Accra and Greater Kumasi) from March 30, 2020. Despite exemption of essential services including health care, hospital attendance reduced for fear of contracting the virus amidst evidence that people with diabetes were at high risk of infection, complication and death. The pandemic led to re-structuring of diabetes services with less contact hours and dwindled staff strength. Regular monthly appointments were adjusted to quarterly to reduce the risk of infection. Travel restrictions affected supply chain shooting up cost of essential medicines and laboratory reagents.

### Research design

This retrospective cross-sectional study involved the use of secondary data extracted from the physical records (folders) of 2,593 patients who presented at the NDMRC at Korle-Bu from 2015 to 2021. Though the facility was founded in 1995 and had over 5000 registered patients as of the time of the study, we utilized patient records from 2015 to 2021 summing up to 2,593 patients (based on year of registration). No multiple information was recorded because authors were interested in estimating the pattern of glycemic control. Inasmuch as there were several visits by some patients, we extracted records based on year of registration of patient with the facility. We utilized the last information of a patient if there were multiple visits in that year. There was no paired data. This approach was appropriate to give a snapshot assessment of glycaemic control throughout the years under consideration, and in light of the COVID-19 pandemic. Data abstraction form was designed based on study objective and patients’ information available. Data was grouped into three categories–Socio-demographic, laboratory and exploratory/anthropometric. Laboratory values were extracted from printed laboratory reports in patients’ folders and crosschecked with physicians’ clinical notes where available. All other data categories were extracted from records as documented in patients’ folders. Folders reviewed and data collected by one researcher were crosschecked randomly by another for data quality and accuracy.

### Outcome variable

The primary outcome variable was Glycaemic Control (GC) which consists of the average measurement of blood glucose (HbA1c). From the raw measurements, three categories were generated based on the American Diabetes Association [17] and the method adopted by Mimenza-Alvarado and colleagues [18] which are in line with diabetes management at the NDMRC. The categories involved were normal (HbA1c < 7%), Intermediate (HbA1c 7–7.9%), and poor (HbA1c ≥ 8%).

Though there has been some controversy on the ideal target for blood glucose control for PLWD on a range of 6.0–7.5% for HbA1c, ADA and the European Association for the study of Diabetes (EASD) concluded that HbA1c goal cut-off point of 7.0% (53.0 mmol/mol) remains optimal [19–21]. The Standards of Medical Care in Diabetes 2022 further iterates that stringent goals of HbA1c results <6.5% (48 mmol/mol) could be set for patients who are deemed capable of attaining such results without the complication of hypoglycaemia or other adverse effects; whilst a less stringent goal of HbA1c value <8.0% (64 mmol/mol) could be set for PLWD with extensive comorbid conditions, history of severe hypoglycaemia and advanced micro-and macrovascular complications such that the benefits of treatment are less than the harms [21, 22] and taking into consideration individual preference of less burdensome therapy or stringent measures of control due to high motivation [21]. These considerations are in sync with diabetes management practice at the NDMRC where treatment plan is individualized for optimal glycaemic control. In our current study, since patients with other chronic conditions such as malignancy, chronic renal failure, chronic liver disease as well as pregnancy were excluded, the HbA1c target of 7.0% (53.0 mmol/mol) or lower considered as good glycaemic control was in place.

### Exposure variable

In this study, authors constructed an exposure-outcome termed, era of COVID-19 pandemic. This variable was generated to assess the COVID-19 pandemic situation in Ghana and its impact on PLWD considering GC. To generate this variable, the authors considered the year in which Ghana experience her index case of COVID-19. The exposure variable was called “era of COVID-19” which was constructed as “Yes = 1” by combing the data for 2020 and 2021 and “No = 0” as otherwise. This procedure was deemed appropriate since the country’s first two cases were reported in 2020 [31].

### Independent variable

Variables considered included sex, age, currently working, Body Mass Index (BMI), Non-high-density lipoprotein (Non-HDL), Low-density lipoprotein (LDL), systolic blood pressure (SBP), and diastolic blood pressure (DBP). BMI was calculated as weight (Kg) divided by height (m^2^) and was categorized as; 18.5–24.9 (normal weight), 25–29.9 (overweight), and 30+ (obesity). Since BMI is adjusted for amputees, a unique category was created under BMI for persons with missing limbs.

### Study population

The study population were patients who attended the NDMRC during the period considered in this study. Socio-demographic characteristics are presented in Table 1. Ages ranged from 12 to 106 years with 0.25% being records of patients who were teenagers (young adults).

**Table 1:** Demographic and clinical characteristics of PLWD by glycaemic control from 2015–2021; evidence from the NDMRC, Ghana.

Variable	Glycaemic control level			Total	Test
	Good	Intermediate	Poor		
%(95%CI)	34.4(32.6–36.3)	14.0(12.8–15.4)	51.6(49.6–53.5)		
	%	%	%	n	
**Sex**					4.65
Man	31.7	14.5	53.8	933	
Woman	35.9	13.8	50.3	1660	
**Age**					10.64***
Mean±SD	56.3±13.4	55.0±12.3	53.7±12.7	54.8±12.9	
**Age group**					18.99**
≤39	33.1	13.1	53.8	329	
40–49	29.0	13.1	58.0	528	
50–59	33.8	14.0	52.1	769	
60+	38.4	14.9	46.8	962	
Missing	20	20	60	5	
**Currently working**					1.07
No	34.7	15	50.3	300	
Yes	34.1	13.8	52.1	1903	
Missing	35.9	14.4	49.7	390	
**BMI**					37.95***
Amputee	29.2	12.5	58.2	838	
Underweight	24.6	21.7	53.6	69	
Normal	33.7	15.0	51.3	493	
Overweight	37.8	12.2	50.0	556	
Obesity	39.9	16.0	44.1	637	
**Non-HDL**					0.67**
Mean±SD	3.7±3.7	3.8±2.8	3.9±2.8	3.8±3.1	
**LDL**					2.31
Mean±SD	3.0±1.3	3.0±1.3	3.1±1.4	3.1±1.4	
**SBP**					33.51***
Mean±SD	135(31)	132(32)	128(38)	131(34)	
**DBP**					27.45***
Mean±SD	79(17)	80(19)	82(21)	80(18)	

NOTE: Abbreviation: BMI = Body Mass Index, HDL = High Density Lipoprotein, LDL = Low Density Lipoprotein, SBD = Systolic Blood Pressure, DBP = Diastolic Blood Pressure, ref = Reference category used for inference. P-value Notation

*p-value<0.05

**p-value<0.01

***p-value<0.001

Data from the National Diabetes Management and Research Centre (NDMRC), Korle Bu Teaching Hospital, Ghana.

### Inclusion/Exclusion criteria

Inclusion criteria was records spanning 2015–2021. Records showing other chronic conditions such as malignancy, chronic renal failure, chronic liver disease as well as pregnancy and inflammatory conditions were excluded.

### Data analysis

Descriptive analysis was adopted for the demographic and clinical variables relating to the objectives of the study. The prevalence of PGC across the years was estimated. For categorical variables (sex, age group, working status, BMI category), chi square (X^2^) analyses were used whiles for quantitative variables (age, Non-HDL cholesterol, LDL-cholesterol, SBP and DBP), one-way analyses of variance (ANOVA) with Bartlett’s test for equal variances used to establish associations with glycemic control. In addition, percentage change within the years and overall increase in rate of PGC were estimated using stock plot analysis. Ordinal logistic regression was adopted to show factors associated with PCG among PLWD. Mahalanobis distance matching was adopted to improve the precision of estimates from the data within propensity caliper to reduce bias estimates by controlling for covariates. In order to reduce imbalances in estimating the effect of COVID-19 era on poor GC, identified factors influencing poor GC were matched to have a precise estimate. This process was deemed fit because it reduced bias in our impact estimation [23–25]. After pre-processing the data with matching, we further employed ordinal logistic and Poisson regression analyses to estimate the impact of COVID-19 era on poor GC by controlling the weighting scores from Mahalanobis distance matching.

All analyses were performed using Stata version 16.1 and P-value ≤ 0.05 deemed significant.

### Ethics statement

Ethical approval was obtained from the Institutional Review Board of the Korle-Bu Teaching Hospital (with Identification Number: KBTH-IRB/00071/2021). Since this was a retrospective study spanning 2015 to 2021, participants could not be reached for their Individual Informed Consent. However, a general consent was obtained from the Head of the Diabetes Unit before beginning data extraction. Confidentiality was strictly observed during and after the study. No identifiable information linking records to patients was included during and after the study to preserve anonymity. Only codes were used to assign participants.

## Results

### Glycaemic control in PLWD and its determinants

**Fig 1:**
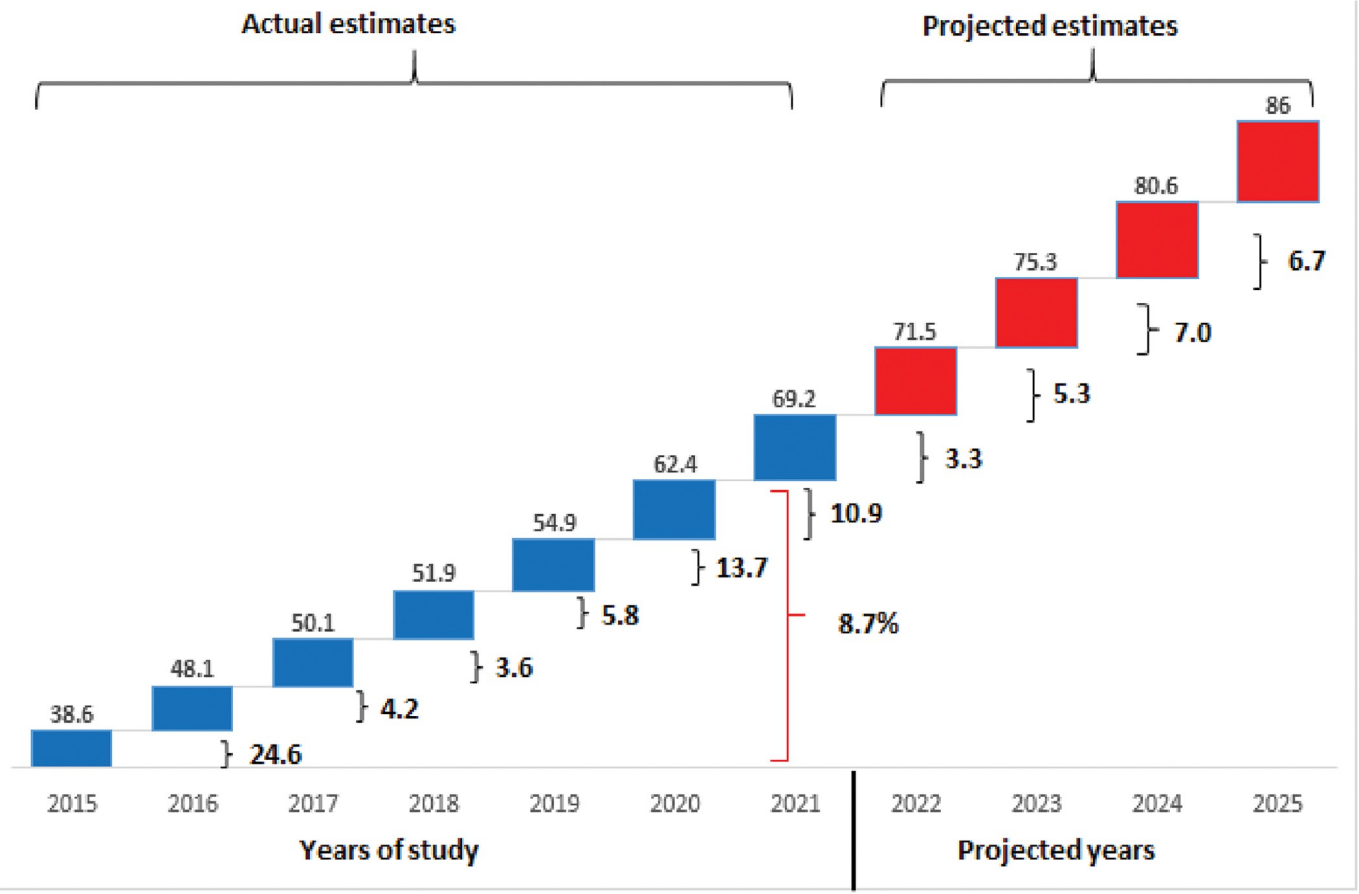
Prevalence, percentage change, and overall growth rate of PGC among PLWD from 2015–2021, Ghana. Figures on top of **BLUE** Boxes indicate actual prevalence rates for poor glycaemic control whilst those in **RED** boxes indicate projected prevalence rates. Figures behind **BLACK** close parenthesis bracket indicates percentage change of poor glycaemic control whilst the figure behind the **RED** indicates the overall growth rate from 2015–2021. Data from the National Diabetes Management and Research Centre, Korle Bu Teaching Hospital, Ghana. PGC = Poor Glycaemic Control.

The study involved 2,593 PLWD ages ranging from 12–106 years with mean±SD of 54.7±12.9 years. In all, women were in the majority, accounting for approximately twice that of men (men versus women was 1:2, respectively). The HbA1C ranged from 4.2–18.9% with mean±standard deviation of 8.87±3.40 and the median (inter-quartile range) was 8.1(4.3). As presented in Fig 1, the trend of PGC increased steadily with a prevalence rate ranging from 38.6% in 2015 to 69.2% in 2021. The overall growth and the percentage change within the years showed a positive rate. The highest percentage increase occurred between 2015 and 2016 with a 24.6% increase rate and the lowest occurred between 2018 and 2019 with a 3.6% increase rate. The pattern of PGC across the years was statistically different (p-value<0.001). The projection also showed an increasing pattern from 2022 with an estimated 86% of PGC at the end of 2025 with all things being equal (Fig 1).

Overall PGC was 51.6% (95%CI = 49.6–53.5) and the differences in proportions and means were statistically associated with age, BMI, Non-HDL, SBP, and DBP (p-value<0.05) (Table 1). Generally, factors including sex differential, age groups, BMI, LDL, SBP, and DBP were significantly associated with PGC.

The analysis depicts that being a woman significantly increases the likelihood of PGC by 22% compared with being a man (aOR = 1.22, 95%CI = 1.01–1.46). Age groups showed that those aged ≤39 years and 40–49 years were 37% and 55%, respectively more likely to experience PGC compared with those aged 60+ years [aOR(95%CI) = 1.37(1.02–1.84) and 1.55(1.22–1.99), respectively]. Increasing LDL and DBP significantly increase the likelihood of PGC by 12% and 25%, respectively [aOR(95%CI) = 1.12(1.03–1.21) and 1.25(1.10–1.41), respectively] whilst increasing SBP significantly decrease the risk of PGC by 19% (aOR = 0.81, 95%CI = 0.71–0.92) (Table 2). As well, age as a risk factor for PGC showed that lower age increased the risk of PGC throughout the various years. Compared with PLWD aged 60+ years, the risk of PGC was high among those aged ≤39 years, 40–49, and 50–59 years, however, was statistically significant in 2016, 2019, and 2021 (Table 2).

**Table 2:** Ordinal logistic regression showing factors associated with PGC among PLWD from 2015–2020; evidence from the NDMRC, Ghana.

Variable	Data extraction period							
	2015	2016	2017	2018	2019	2020	2021	Pooled
	aOR[95%CI]	aOR[95%CI]	aOR[95%CI]	aOR[95%CI]	aOR[95%CI]	aOR[95%CI]	aOR[95%CI]	aOR[95%CI]
**Sex**								
Woman	**Ref**	**Ref**	**Ref**	**Ref**	**Ref**	**Ref**	**Ref**	**Ref**
Man	1.38[0.92–2.08]	1.07[0.68–1.68]	1.45[0.98–2.13]	0.85[0.48–1.48]	1.17[0.68–2.01]	1.01[0.57–1.79]	0.80[0.47–1.33]	1.22[1.01–1.46]*
**Age group**								
60+	**Ref**	**Ref**	**Ref**	**Ref**	**Ref**	**Ref**		
≤39	1.92[0.97–3.79]	1.68[0.85–3.35]	1.30[0.71–2.38]	1.19[0.45–3.12]	1.44[0.62–3.37]	1.20[0.43–3.32]	1.33[0.58–3.04]	1.37[1.02–1.84]*
40–49	1.45[0.82–2.56]	2.34[1.25–4.36]**	1.46[0.90–2.39]	0.99[0.47–2.10]	2.79[1.30–6.00]**	1.78[0.78–4.10]	1.65[0.81–3.37]	1.55[1.22–1.99]***
50–59	1.04[0.62–1.75]	2.17[1.25–3.75]**	1.38[0.87–2.19]	0.51[0.26–0.98]*	1.42[0.76–2.66]	1.70[0.83–3.48]	1.85[0.97–3.48]	1.25[1.01–1.56]*
**Currently working**								
Yes	**Ref**	**Ref**	**Ref**	**Ref**	**Ref**	**Ref**		
No	1.20[0.57–2.53]	1.41[0.66–3.05]	1.13[0.59–2.15]	0.37[0.16–0.83]*	1.83[0.93–3.60]	1.03[0.56–1.91]	0.63[0.27–1.45]	1.20[0.92–1.56]
Missing								
**BMI**								
Normal	**Ref**	**Ref**	**Ref**	**Ref**	**Ref**	**Ref**		
Amputee	1.22[0.70–2.12]	0.98[0.49–1.96]	0.74[0.42–1.28]	1.26[0.42–3.82]	0.75[0.35–1.62]	0.84[0.37–1.91]	2.01[0.78–5.17]	1.00[0.77–1.29]
Underweight	2.23[0.46–10.8]	0.57[0.17–1.91]	1.59[0.39–6.49]	0.91[0.22–3.83]	5.18[0.58–45.90]	1.77[0.17–18.77]	-	1.36[0.76–2.42]
Overweight	0.86[0.49–1.52]	0.88[0.46–1.68]	0.81[0.48–1.37]	0.61[0.31–1.22]	0.86[0.43–1.71]	1.57[0.49–5.00]	-	0.85[0.66–1.10]
Obesity	0.76[0.42–1.35]	0.54[0.29–1.01]	0.71[0.42–1.17]	0.89[0.44–1.80]	0.71[0.36–1.40]	0.65[0.25–1.72]	-	0.75[0.59–0.97]*
**Lipid & BP**								
Non HDL	3.14[1.73–5.69]***	0.98[0.42–2.31]	1.11[0.60–2.07]	2.69[1.05–6.91]*	0.92[0.41–2.03]	0.68[0.32–1.44]	0.97[0.92–1.02]	1.00[0.96–1.04]
LDL	0.35[0.19–0.66]***	1.23[0.49–3.12]	0.92[0.47–1.81]	0.41[0.14–1.16]	1.15[0.49–2.69]	1.70[0.79–3.70]	1.03[0.88–1.22]	1.12[1.03–1.21]**
SBP	0.85[0.64–1.13]	0.69[0.50–0.94]**	0.84[0.65–1.10]	0.96[0.67–1.39]	0.96[0.67–1.39]	0.55[0.36–0.84]**	1.09[0.32–3.62]	0.81[0.71–0.92]***
DBP	1.23[0.93–1.61]	1.43[1.03–1.97]*	1.09[0.83–1.44]	1.25[0.86–1.81]	0.92[0.64–1.30]	2.03[1.32–3.11]***	0.90[0.21–3.81]	1.25[1.10–1.41]***

NOTE: Abbreviation: aOR = Adjusted Odd Ratio, BMI = Body Mass Index, HDL = High Density Lipoprotein, LDL = Low Density Lipoprotein, SBD = Systolic Blood Pressure, DBP = Diastolic Blood Pressure, ref = Reference category used for inference. P-value Notation

*p-value<0.05

**p-value<0.01

***p-value<0.001

Data from the National Diabetes Management and Research Centre (NDMRC), Korle Bu Teaching Hospital, Ghana.

### Glycaemic control in COVID-19 era

**Fig 2:**
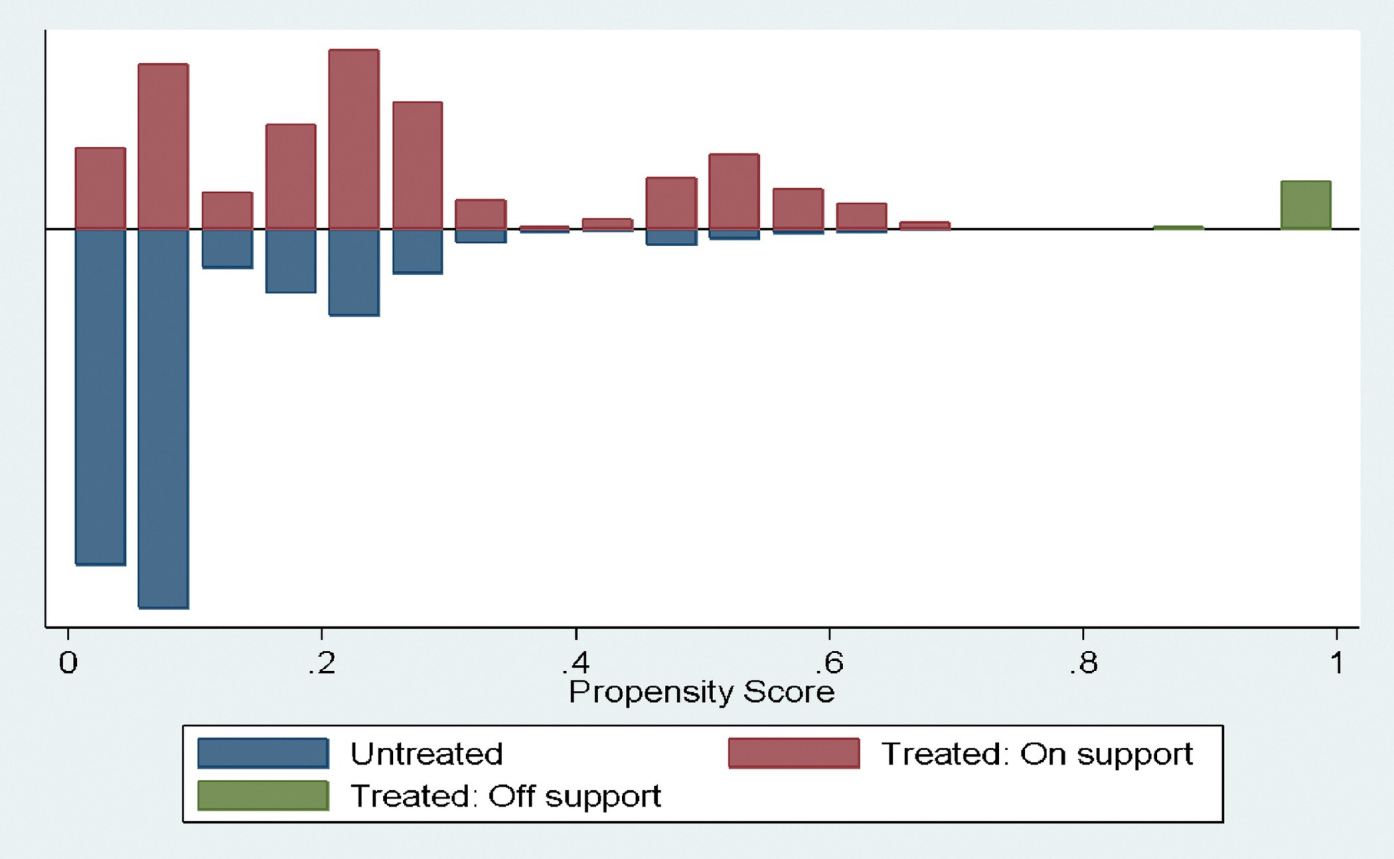
Assumption of common support as assessed by propensity matching method.

Common support assumption as presented in Fig 2 clearly depict an overlap of the propensity scores between the era of COVID-19 and that of the previous years without COVID-19 pandemic.

**Fig 3:**
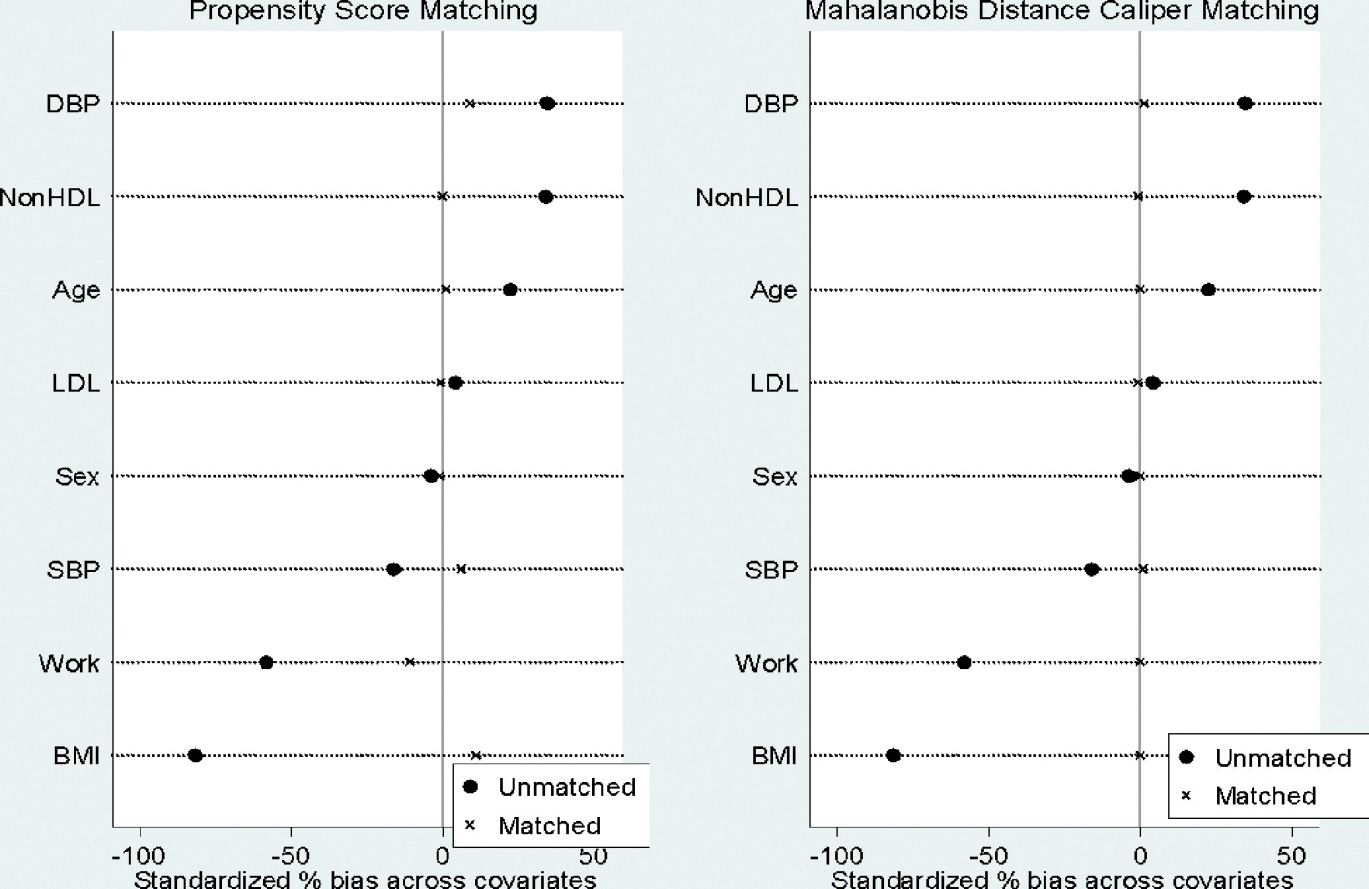
Comparing propensity score matching and Mahalanobis distance within propensity score showing standardize percentage bias across covariates.

To improve the precision of estimates from the data, the authors adopted the Mahalanobis distance matching within propensity caliper to reduce bias estimates by controlling for covariates. The matching procedure clearly demonstrates that the normal propensity score matching showed some covariates not achieving approximately 0% standardized bias, however, after adoption of Mahalanobis distance matching within propensity caliper, there was approximately 0% standardized bias matching between covariates (Fig 3).

After controlling Mahalanobis distance within Propensity Score caliper weights, estimates showed that there was an increased risk of PGC during the era of COVID-19 pandemic among PLWD. Analysis showed that the risk of PGC during the era of COVID-19 was approximately 1.57 times significant compared with the era without COVID-19 (aOR = 1.57, 95%CI = 1.08–2.30). Meanwhile, artificial binary outcome of PGC showed that the adjusted prevalence ratio (aPR) of PGC during the era of COVID-19 was approximately 64% significantly higher than the era without COVID-19 (aPR = 1.64, 95%CI = 1.10–2.43). In addition, Logistic and Probit predicted an adjusted Odds and log count of 1.22 times (95%CI = 1.01–1.47) and 0.30 coefficient (95%CI = 0.06–0.54), respectively (Table 3).

**Table 3:** Influence of COVID-19 era on PGC among PLWD from 2015–2020; evidence from the NDMRC, Ghana.

Variable	Main outcome	Artificial Outcome-Binary nature		
	Ordinal Logistic	Poisson	Logistic	Probit
	aOR[95%CI]	aPR[95%CI]	aOR[95%CI]	aβ[95%CI]
**Era of COVID-19**				
No	**Ref**	**Ref**	**Ref**	**Ref**
Yes	1.57[1.08–2.30]**	1.64[1.10–2.43]**	1.22[1.01–1.47]*	0.30[0.06–0.54]**
**Sex**				
Woman	**Ref**	**Ref**	**Ref**	**Ref**
Man	1.49[0.98–2.28]	1.34[0.86–2.09]	1.12[0.92–1.36]	0.18[-0.09–0.45]
**Age group**				
60+	**Ref**	**Ref**	**Ref**	**Ref**
≤39	1.03[0.45–2.35]	1.01[0.44–2.34]	0.97[0.70–1.35]	0.01[-0.50–0.52]
40–49	0.90[0.48–1.69]	0.89[0.46–1.71]	0.95[0.73–1.23]	-0.07[-0.46–0.33]
50–59	0.78[0.45–1.37]	0.79[0.44–1.41]	0.90[0.69–1.18]	-0.14[-0.50–0.21]
**Currently working**				
Yes	**Ref**	**Ref**	**Ref**	**Ref**
No	0.61[0.37–1.00]	0.53[0.31–0.90]**	0.75[0.56–1.00]*	-0.39[-0.71–0.06]
Missing				
**BMI**				
Normal	**Ref**	**Ref**	**Ref**	**Ref**
Amputee	0.94[0.52–1.69]	1.22[0.65–2.30]	1.10[0.83–1.45]	0.12[-0.27–0.50]
Underweight	0.78[0.16–3.82]	0.76[0.13–4.38]	0.90[0.39–2.06]	-0.18[-1.27–0.91]
Overweight	1.15[0.52–2.53]	1.44[0.63–3.30]	1.16[0.84–1.60]	0.22[-0.29–0.73]
Obesity	0.59[0.29–1.18]	0.60[0.28–1.27]	0.80[0.56–1.14]	-0.33[-0.79–0.13]
**LIPID & BP**				
Non HDL	1.46[0.76–2.80]	1.54[0.80–2.98]]	1.19[0.91–1.54]	0.26[-0.13–0.65]
LDL	0.71[0.36–1.40]	0.69[0.35–1.39]	0.87[0.65–1.15]	-0.22[-0.63–0.19]
SBP	0.97[0.96-.99]***	0.98[0.96–0.99] ***	0.99[0.98–1.00] ***	-0.01[-0.02-.01] ***
DBP	1.05[1.02-.07]***	1.04[1.01–1.07] ***	1.02[1.00–1.03] **	0.2[0.01–0.04] ***

NOTE: Abbreviation: aOR = Adjusted Odd Ratio, BMI = Body Mass Index, HDL = High Density Lipoprotein, LDL = Low Density Lipoprotein, SBD = Systolic Blood Pressure, DBP = Diastolic Blood Pressure, ref = Reference category used for inference. P-value Notation

*p-value<0.05

**p-value<0.01

***p-value<0.001

Data from the National Diabetes Management and Research Centre (NDMRC), Korle Bu Teaching Hospital, Ghana

## Discussion

### Glycaemic control in PLWD and associated factors

This study confirms the increasing proportion of PGC among PLWD. Additionally, PCG further worsened in the COVID-19 era. Aschner et al. [8] revealed similar findings in their observational study in developing nations. They found that glycaemic control in people with type 2 diabetes remained poor, emphasizing the need for system improvements and improved quality of care to enhance self-management and treatment objectives [8].

In an institutional-based cross-sectional study by Fekadu et al. [26], a similar trend of poor GC was detected, with two-thirds of their study population showing poorly controlled diabetes. However, compared with other studies, findings established in this current work appear relatively lower. Chetoui and colleagues found a PGC prevalent rate of 66.3% among T2D in the Moroccan population in 2017 [27]. These findings could be due to the different geographical location of participants and the study design involved. The current study adopted a retrospective approach whilst the above literature employed a cross-sectional technique. In Ghana, a prospective cross-sectional study conducted in 2018 also found higher results with PGC accounting for 69.7% as compared with 51.9% in this current study [7]. In the same year, a 5-multi-centre-Ghanaian study involving 1226 participants [28] found the PGC prevalence rate of 70%. Findings from earlier and later years have not been different.

PGC levels, such as found in this study, remain a significant sequela for complications of diabetes. As presented in Fig 1, approximately one-third and two-thirds of PLWD had PGC in 2015 and 2021, respectively, which clearly explains the 8.7% growth rate of PGC. Whilst the COVID-19 pandemic may have contributed to worsening of glycaemic control, the growth rate observed from 2015 to 2021 signals a need to revisit interventions towards diabetes control and management. Though COVID-19 may have interrupted diabetes services at the centre and across the country, an increasing pattern of PCG was observed within the study period. We project that if nothing is done to arrest the observed pattern, the prevalence of PGC may reach a record high of 86% by the end of 2025.

### Influencers of glycaemic control among PLWD

Being a woman significantly increased one’s likelihood of having PGC. This finding is consistent with Mobula et al., 2018 study [28] in Ghana where men were 34% more likely to have good glycaemic control than women. Similarly, in a study focused on developing countries in seven cross-sectional waves from 2005 to 2017, patients with diabetes who were younger and were women, were also less likely to achieve the HbA1c goal compared with their counterparts [8]. Sex has been found to be a significant determinant in the storage of lipids in muscles and liver, and is a predictor of metabolic activity and development of metabolic disease [29]. Since women rely more on fat reserves as fuel during physical activity (PA) than men [29, 30], the latter could better control their fat and muscle reserve and thereby reduce their BMI. In fact, PA is a significant determinant of PGC [7, 31–33]. Meanwhile, PA may be less in women than men [7, 34, 35] inasmuch as there is difference in PA preference and motivating factors across both sexes [30, 36]. It has been found that PA (even of light intensity), reduces postprandial glucose and insulin levels as compared to a sedentary lifestyle. PA also points to improved cardiometabolic health and reduced risk of mortality [37]. Amidst all odds, COVID-19 has been linked to depression, more in women than men [38]. With the link between depression and PGC [39], and the two-year astronomical increase in PGC in 2020 and 2021 observed in the current study, we believe these are probable influencers of PGC among women compared to men. In addition, clinics were disrupted in the first year of the pandemic, and many patients for fear of infection, stayed away. Indeed, patients were given longer review dates than usual. We advocate for a system of follow up, especially on high-risk patients to mitigate the impact of service interruption that was observed.

Being of younger age is linked to PGC as well. This translates to ages below 60 years being associated with PGC. The current finding is not different from that of significant others that pointed out age as a notable determinant of glycaemic control [28, 31, 33]. We argue that older people tend to easily adhere to treatment modalities and diabetes management plans. Being relatively older could be linked to having had enough life experiences thereby reducing the youthful, hectic stress-packed lifestyle. Older patients may be more motivated to control their diabetes, more likely to be medication compliant as well as stick to a more balanced diet free of high carbohydrates. On the other hand, persons of younger age may be less likely to adhere to medications, make lifestyle changes and nutritional adjustment. Change in general lifestyle, occupational and social life, thereby tend to relatively affect people of the younger generation [31], whilst the wake of comorbidities and complications among older people [40] increase their tendency to take charge and control their health thereby improving glycaemic control.

Selvin and Parrinello [41] presented elaborate review on why PGC may be more prevailing at younger-age compared to older age. They explained that the pathophysiology of type 2 diabetes is different in the elderly compared to that of younger age; as opposed to insulin resistance which mostly leads to diabetes at younger age, impaired insulin secretion is implicated in the elderly [42] which may affect the effectiveness of some medications such as metformin in the elderly [43]. Persons of younger ages have also been found to present with severe form of the disease along with complication such as obesity, higher degrees of insulin resistance and rapidly increasing blood glucose [44] which may explain the high use of insulin in this group compared to sulphonylurea medications [45]. Finally, Selvin and Parrinello [41] described how survival bias may be a contributing factor to the apparent better GC observed in the elderly. They argued that many elderly with PGC may have “fallen off” early along the way and hence not included in many cross-sectional studies. State of frailty, comorbid conditions, and being on multiple medications among others, often render the elderly ineligible for epidemiological studies and clinical trials.

We also found that increasing low-density lipoprotein cholesterol (LDL) but not triglyceride (TG), total cholesterol (TCHOL), and non-high-density lipoprotein (non-HDL) significantly increased the likelihood of PGC. This is in contrast to studies by Naqvi et al and Hinton et al. [46, 47] which found glycaeted haemoglobin to correlate positively with triglyceride. Other studies have found dyslipidemia [48] with TCHOL [46] as predictors of PCG. Dyslipidemia is elevated levels of LDL or TCHOL or low levels of HDL or both [49]. Inasmuch as one or more abnormalities in the serum lipids signifies dyslipidemia [48] more frequently and operationally, LDL cholesterol from most laboratories is a derived parameter from TCHOL, TG, and HDL. Therefore, the significance of LDL in the current study of relatively large data implicates the combined effect of lipid parameters in influencing glycaemic control. Clinicians must not only focus on glycaemic control but must tackle all cardiovascular risk factors including dyslipidemia. As dyslipidemia significantly increases one’s risk of coronary heart disease and stroke [49], its control remains paramount in patients with diabetes for primary and secondary prevention of cardiovascular disease.

Interestingly, we found that increasing diastolic blood pressure (DBP) is significantly associated with PGC—a finding similar to a study by Torchinsky et al. [50]. They found significant positive correlation between HbA1c and DBP. However, their study population was among children with type 1 diabetes [50]. Increasing systolic blood pressure (SBP) was found to be associated with a decreased risk of PGC. While we do not suggest that high SBP is a protective factor for good glycaemic control, the possibility that persons with elevated SBP attending the clinic regularly are already on stringent hypertension management could account for this finding. Despite the control of all three targets of HbA1c, lipids, and BP among PLWD remaining suboptimal, BP remains the poorest achievable target [51]. It is argued that inasmuch as some diabetes medications can affect blood pressure, the dynamics may not be the same across all diabetes population [52]. That notwithstanding, the proclivity to focus on SBP in predicting a patient’s risk for cardiovascular events and hence commencing antihypertensives may result in implications for DBP going unnoticed. This is because PLWD with relatively lower SBP may be overlooked whilst their DBP remains indicative of PCG.

According to Van der Merwe [53], baseline DBP of less than 80 mmHg in those under the age of 50 is a considerably better predictor of future hypertension than baseline SBP of more than 120 mmHg. Huang et al. [54] also emphasized how isolated diastolic hypertension is frequently disregarded and its awareness still low despite the United States and some countries in Asia appear to be experiencing an upsurge in prevalence [54]. Aging has been cited as a key factor in hypertension management. A study by Pinto [55] validated the trend of DBP to vary with age, increasing until the fifth decade and progressively declining from the age of 60 to at least 84 years. Our population being a relatively younger one (mean age being 54.7 years) could also explain our finding. In the same way, patients with higher BMI (obese) may present with more complications and co-morbidities and hence may be put on more stringent treatment modalities to effectively control their blood glucose. This could possibly account for the observed relationship between obesity and PGC in our study. This was the case where the type of regimen such as being on metformin, insulin, and combination of insulin and oral-antidiabetic drugs, as well as having cardiovascular disease were found to influence good glycaemic control [7, 27, 31, 32]. We therefore recommend combined use of SBP and DBP in blood pressure control, especially in DM management.

Sendekie et al. [56] suggest high SBP as a significant determinant of PGC. They reported that after initiation of insulin therapy, many PLWD who had high SBP, did not achieve optimal glycaemic control after twelve months. Notably, in their study, the effect of DBP was not explored or presented. This confirms our assertion on the seeming neglect of DBP in many instances. That notwithstanding, their study involved switching from other treatment guidelines to solely insulin as opposed to our study that involved patients who were on either medications or insulin or combinations.

Concomitant high BP and diabetes have been linked to arterial stiffness that facilitates hypertension through a gradual breakdown and loss of elastin fibers in blood vessels, contributing to the development of chronic kidney disease and stroke [57]. However, some combinations of antihypertensive in diabetes may not be ideal and should be carefully thought through by clinicians in diabetes management [57, 58]. In addition, with the increasing argument on ideal target of BP control in T2DM (whether targeting 130/80mmHg or SBP <120mmHg) such that cardiovascular complications do not set in [59] and the relevance of combined BP and glycaemic control in diabetes, treatment and control should be individually tailored to achieve utmost effectiveness and efficiency whilst we work towards newer therapeutic strategies.

### Glycaemic control in the era of COVID-19

In providing a guideline for assessment and management of diabetes in COVID-19, Verma et al. [60] pointed out that the dysregulated immune response in DM remains a significant driver of having COVID-19 disease severity. However, it remains unclear the effect of the pandemic on diabetes. Our impact evaluation analysis showed that there was an increased risk of PGC during the era of the COVID-19 pandemic among PLWD as per records abstracted from the NDMRC of the Korle-bu Teaching Hospital.

Inasmuch as the highest percentage increase in PGC occurred between 2015 and 2016 with a 24.6% increase rate, we observed that the rate of increase in the ensuing years till 2019 was significantly lower with rates of 4.2%, 3.6%, 5.8% recorded in 2017, 2018 and 2019, respectively. Considering the period of our study, we are unable to comment on the rate of PGC hitherto 2015. However, although the rates have been steadily rising after 2016, we can speculate from the trend observed that small gains were being made toward reducing the prevalence of PCG until the steep rise observed in 2020. We found approximately 3 times increase in the percentage change in PGC in 2020 and 2021 when COVID-19 wreaked havoc on health systems the world over. The risk of PGC during the peak era of the pandemic was approximately twice significant compared with the period before.

Key explanation for this significant finding could be that the pandemic era has greatly affected diabetes management [61] in essence that the situation reduced contact with health professionals, led to medication stock outs, and probable consequence of lockdown periods playing a role in PGC as observed in the current study. The high levels of psychological distress seen during the era of COVID-19 could be a contributing factor to PGC as stress has been linked to PCG [62].

A possible new diagnosis of diabetes may as well have impacted on the prevalence of PGC as authors observed in the current study. We recommend holistic public health promotion strategies and policies aimed at controlling irrational and unhealthy eating, enhancing PA, promoting mental wellbeing as well as inculcating intentional and sustained efforts towards maintaining healthy BMI among youths and the general populace. Again, continuous efforts toward control of COVID-19 will have translational benefits on diabetes control. The impact of COVID-19 era on GC is a key issue to be factored in public health interventions.

### Limitations

A major limitation of this study is that the results of laboratory data extracted were not from one laboratory. Test methods amongst these labs may vary according to equipment and reference ranges used. Some folders were missing and amongst those available, some had missing responses. Diabetes was considered across board and not categorized into Type 1 or Type 2, although majority appeared to be Type 2 as only 0.25% of data extracted were of persons under 20years.

Comorbid cardiac disease was not ruled out. An HB_A_1c that is considered poor may be considered appropriate in patients with comorbid cardiac disease. Moreover in elderly patients, less stringent targets are often used because the harms of such targets may outweigh the benefits. Generalizability of our data must be interpreted within the context of the study.

Additionally, the utilization of secondary data in this study presented the challenge of incomplete data on some specific variables (such as regarding the comorbid situation of all patients, alcohol-use, smoking status, etc.) whose impact we may have explored.

Our projection of PGC was based on available data and prevailing circumstances of COVID-19 pandemic in 2020 and 2021. We believe that the pandemic era greatly affected diabetes management in terms of reducing contact with health professionals, medication stock outs, and the probable consequences of lockdown among other factors. High levels of psychological distress during the pandemic era, as well as new diagnosis, could also be implicated. If all these factors are normalized or neutralized by effective standards and controls, the outcome may not be as dire. That notwithstanding, the pandemic era contributed only a year to the projection made compared to the 6 years without it–thereby improving the strength of our evaluation.

## Conclusion

The trend and pattern of glycaemic control appears to worsen over the period 2015 to 2021. PCG was found to be significantly linked with sex, age, LDL cholesterol and blood pressure. The risk of experiencing PCG amongst PLWD increased significantly during the era of COVID-19. The National Diabetes Management and Research Centre and other centres that provide specialist healthcare in resource-limited settings, must determine the factors that militated against optimum service delivery in the era of the COVID-19 pandemic, and implement measures that would improve resilience in provision of essential care in the face of shocks.

## Supporting information

S1 Data Data underlying the manuscript—Changes in trends and patterns of glycaemic control at Ghana’s National Diabetes Management and Research Centre during the era of the COVID-19 Pandemic. (XLSX)

S2 Data Data underlying the manuscript—Changes in trends and patterns of glycaemic control at Ghana’s National Diabetes Management and Research Centre during the era of the COVID-19 Pandemic. (DTA)
